# Personalized Antiarrhythmic Therapy Using a Self-Managed Daily-ECG Device

**DOI:** 10.3390/diagnostics13182864

**Published:** 2023-09-05

**Authors:** Eugenio Mattei, Stefano Lino, Federica Censi, Giovanni Calcagnini, Leonardo Calò

**Affiliations:** 1 Department of Cardiovascular, Endocrine-Metabolic Diseases and Aging, Istituto Superiore di Sanità, 00161 Rome, Italy; eugenio.mattei@iss.it (E.M.); giovanni.calcagnini@iss.it (G.C.); 2 Department of Cardiology, Policlinico Casilino, 00169 Rome, Italy; slino.polcas@eurosanita.it (S.L.); leonardocalo.doc@gmail.com (L.C.)

**Keywords:** atrial fibrillation, daily ECG device

## Abstract

A 50-year-old Caucasian man arrived at the emergency department presenting paucisymptomatic atrial fibrillation. Once discharged after the appropriate treatments, the patient continued to have paucisymptomatic episodes. For this reason, he was provided with the Cardionica device which made it possible to better investigate the type of arrhythmic episodes, in order to tailor his therapy and to finally restore a normal sinus rhythm in the patient.

## 1. Introduction

Atrial fibrillation (AF) is the most common sustained arrhythmia worldwide, and it is the arrhythmia that most often requires hospitalization of the affected patient [1]. The data relating to the overall prevalence of the disease indicate values of up to 4% in the adult population. While not a lethal arrhythmia, AF is associated with stroke, heart failure, cardiomyopathy, and many other medical comorbidities, including death [1,2,3]. The optimal strategy to manage this disease continues to be a challenge for clinicians. Currently, the treatment of AF includes anticoagulation, rate control, and rhythm control [2]. Ventricular rate control is an established therapy, and it is now widely accepted as an alternative to rhythm control for first-line management of chronic AF and rapid ventricular response [3]. There are important reasons to control the ventricular rate in patients presenting with AF, such as the patient’s symptomatic status, hemodynamic instability and risk of developing cardiomyopathy. It has been demonstrated that rate control therapy may have a lower risk of adverse drug toxicities without affecting overall mortality [3]. Rate control strategy choice is very specific to each individual patient, since one size does not fit all patients. Indeed, the choice of the drug and relative dosage will depend on the patient’s characteristics, occupation, age, ability to tolerate medications, degree of symptoms, degree of functional limitation, left ventricular ejection fraction value, hemodynamics, and heart rate [2]. Usually, a lenient initial rate control approach seems acceptable [1]. Then, dose adjustments based on close monitoring of the heart rate and/or ECG should be performed to achieve adequate rate control [3]. Drug doses in general should be titrated to patient well-being, taking into account the heart rate at rest and during activity and potential adverse effects from treatment. 

In a non-clinical environment, the monitoring of heart rate and/or ECG monitoring is not straightforward, and thus the drug dosage can take several days or weeks before being optimized. In this context, a self-managed daily ECG can help in monitoring the cardiac patient’s status, in terms of heart rate and ECG. Modern technology now offers various devices that allow the recording of a one-lead ECG signal, made directly by the patient, at any time and in any place [4,5]. Some of these devices offer a real-time diagnosis of AF, bradycardia and tachycardia, while others record an ECG trace which must then be sent to the doctor for reporting. All devices allow the sharing of data or of a report of results. Many of these devices record an ECG from the fingers, while others use electrodes as sensors. These devices have been widely studied and are currently mainly used for the screening and diagnosis of AF [5,6]. 

**Figure 1:**
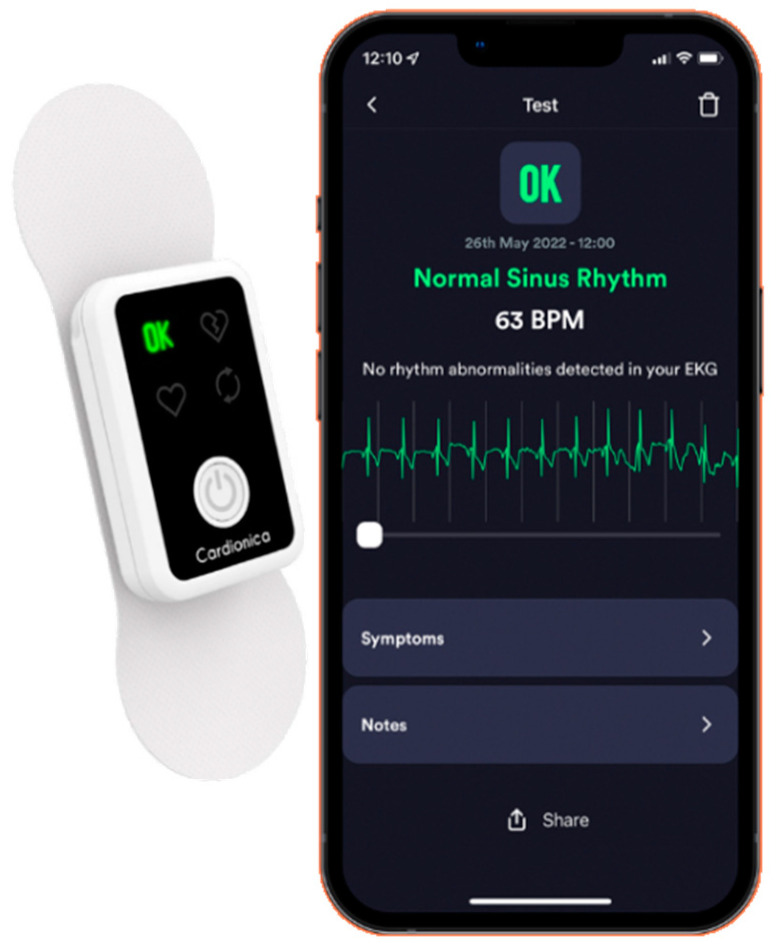
The Cardionica device.

The Cardionica is a brand-new device, intended to be used daily by a re-usable ECG electrode to check for the presence of arrhythmias such as atrial fibrillation, tachycardia and bradycardia (Figure 1). The Cardionica is easy to use and provides a medical grade ECG. 

We hereby describe a case report of a patient diagnosed with AF for whom the Cardionica was decisive in optimizing their therapy.

## 2. Materials and Methods

The Cardionica (MIR Medical Internationa Research SpA, Roma, Italy) is a medical device which detects episodes of atrial fibrillation when placed on the thorax by an adhesive electrode. It is intended for use by healthcare professionals and by any adult subject who wants to monitor atrial fibrillation episodes on an intermittent basis. The device acquires a 1 min-long ECG signal from one adhesive and re-usable electrode and processes the signal in real time. It automatically and autonomously detects episodes of atrial fibrillation, tachycardia and bradycardia, and it can be used by the patient frequently (one or more times a day), increasing the possibility to detect arrhythmic episodes. The Cardionica can be used either as a stand-alone device or in connection with a Bluetooth smartphone/tablet (via a free App, available for both Android and iOs). In stand-alone mode, the Cardionica provides 4 types of response using color indicators: a red heart icon in the presence of an AF episode, a yellow heart icon in the presence of tachycardia or bradycardia, a green ‘OK’ icon in the absence of an AF episode and a yellow double arrows icon if it cannot perform the analysis. When used in app-mode, the device shows the ECG trace and the response of the analysis on the smartphone/tablet screen. The app also allows the downloading of data of tests performed in stand-alone mode and enables the ECG traces available as an edf file to be shared. In addition, a pdf report can be generated and shared for each test. 

With respect to the currently available medical-grade consumer ECG devices, the Cardionica device has 2 novel characteristics. First, it is the only device to use a reusable ECG electrode. The use of an ECG electrode always guarantees acceptable recording quality compared to what happens in devices that use the fingers, in which the quality of the ECG trace is often poor and leads to inconclusive tests [4]. Also, the reusability of the electrode increases the usability of the device, as the disadvantage related to the supply of single-use electrodes is eliminated. Secondly, the Cardionica can be also used in stand-alone mode, and not only in combination with an app, making it particularly suitable for patients who are not particularly accustomed to the use of digital technologies, such as elderly patients.

## 3. Case Presentation

In September 2021, a 50-year-old Caucasian man (height 180 cm, weight 100 kg) arrived at the emergency department presenting atrial fibrillation with a paucisymptomatic high ventricular response (150 bpm) of undated onset and severe left ventricular dysfunction (EF < 25%), NYHA class 3. The diagnostic hypothesis was congestive heart failure (CHF) caused by tachycardiomyopathy, and thus the patient was admitted to the coronary intensive care unit with infusion diuretic therapy and therapy for heart failure (ARB and then ARNI, beta-blockers, antialdosterones and anticoagulants). Electrical cardioversion was not attempted at the admission, since the onset of AF was not known.

In the following days, there was an improvement in heart failure and a reduction in the mean ventricular response to AF (about 95 bpm). Coronary angiography showed no significant epicardial vessel disease.

Since the transesophageal echo examination showed no thrombi, two cardioversions at 200 J were attempted, after conditioning with amiodarone, which proved to be ineffective. Subsequently, the presence of fibrosis was excluded by performing an MRI examination. The patient was then transferred to the rehabilitation unit, and he was discharged after 30 days. At discharge, the patient had AF with a ventricular response of 80 bpm. The therapy on discharge included ARNI, beta-blockers, antialdosterones, anticoagulants and amiodarone in view of a possible subsequent attempt to restore the sinus rhythm. At follow-up visits, spontaneous recovery of sinus rhythm was found with an average heart rate of 65 bpm. No noteworthy changes in ventricular repolarization were found on the ECG. Improved LV dysfunction from previous values persisted on the echocardiogram, with an EF of approximately 40%. Subsequently, paucisymptomatic episodes of elevated heart rate detected during self-measurement of blood pressure were reported by the patient to the treating cardiologist. In order to diagnose the type of arrhythmic episodes, the patient was provided with the Cardionica device with instructions to perform ECG recordings twice a day or in the presence of symptoms.

**Figure 2:**
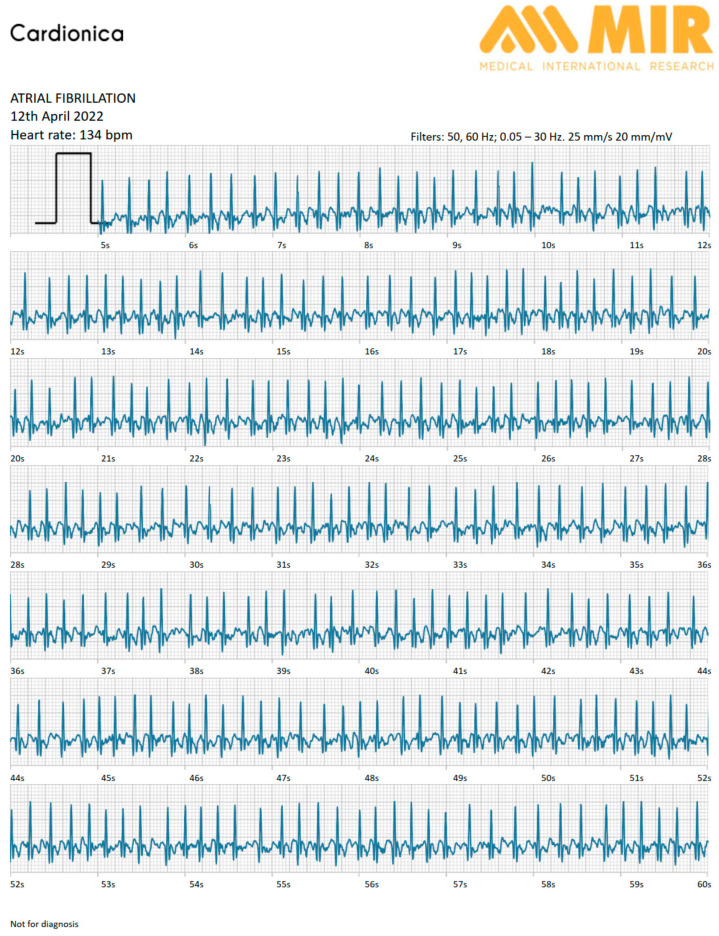
Report of the Cardionica device during the monitoring time, showing atrial fibrillation with high ventricular response.

The patient was asked to use the device in stand-alone mode until the next follow-up visit, for a total of 6 days. The patient followed the doctor’s instructions, making at least 2 recordings a day. On some days, he also used it in the presence of symptoms, for a total of 15 recordings. After 6 days, at the follow-up visit, the cardiologist checked all the recordings and found intermittent episodes of atrial fibrillation with an average ventricular response of 140 bpm (Figure 2). All ECG recordings were of excellent quality and suitable for making a diagnosis.

**Figure 3:**
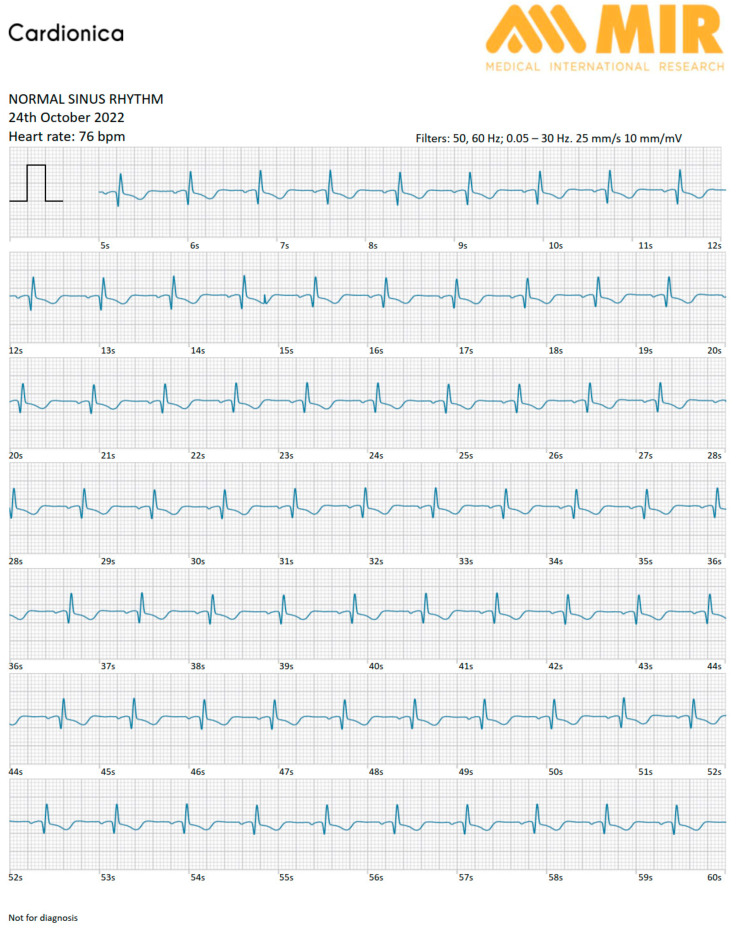
Report of the Cardionica device after the tailoring of the therapy, showing normal sinus rhythm.

The cardiologist then decided to titrate the beta-blocker therapy. This dose optimization allowed the cardiologist to obtain better control of the average ventricular response during the arrhythmic episodes, which were later resolved (Figure 3). Table 1 reports the medications and their doses from admission until the last follow-up visit; gray cells indicate the titration of the beta-blocker therapy after the use of the Cardionica device.

The patient is currently in sinus rhythm and no ablation procedure has been planned, since he is well managed with drug therapy.

## 4. Discussion

The scientific literature has widely investigated the effectiveness of daily ECG monitoring to detect atrial fibrillation [6]. To our knowledge, this is the first work on the use of the daily ECG to optimize antiarrhythmic therapy.

This clinical case demonstrates that the Cardionica has been decisive for establishing the type of arrhythmia of the patient and the correct therapy. Drug dosage optimization could not initially be performed in the patient because of the lack of a clear clinical ECG situation.

The use of the holter as an alternative solution for the diagnosis of arrhythmic episodes would probably not have led to such a result, unless an at least 7-day holter was used, with higher costs and less comfort for the patient. Similarly, the use of a loop recorder would have been much less comfortable and more expensive, also in consideration of the fact that the Cardionica can be used for any number of days, until the diagnosis has been made. Other currently available medical-grade consumer ECG devices would have allowed daily ECG monitoring. However, these devices mainly rely on taking the ECG signal from the finger, which often leads to recordings of such poor quality that it is impossible to make a diagnosis [4]. The use of an ECG electrode, instead, always guarantees an excellent quality of signal. Moreover, the reusability of the electrode has a positive impact on the ease of use and leads to more affordable ECG monitoring. In addition, many, if not all, consumer ECG devices must be used in conjunction with an app and therefore require a certain patient involvement and familiarity with digital technologies that patients may or may not have. The use of stand-alone devices makes monitoring less demanding for patient with limited technological ability, as they only have to switch on the ECG device without having to start a smartphone or tablet, download and install an app and to send the tracing data to the doctor.

The Cardionica allowed to the cardiologist make a diagnosis and to safely optimize drug therapy using only one electrode, providing ECG traces of good quality and without any discomfort for the patient who used the device in stand-alone mode with no difficulty.

The presented case opens the way for the possibility of using ECG personal use devices for purposes other than the identification of arrhythmias, also providing help in tailoring the therapy.

## 5. Conclusions

Cardionica has proved to be an ideal device for personalized medicine, being economical and without discomfort for the patient. Furthermore, beyond this clinical case, its enormous competitive advantage also lies in its ability to perform self-diagnosis of atrial fibrillation, thus allowing the diagnosis of this intermittent and often asymptomatic pathology and in general the monitoring of AF episodes even in patients under antiarrhythmic and/or anticoagulation therapy. This case report shows the practicality and usefulness of monitoring devices that allow easy access to the patient and the doctor: the more information there is available, the better the treatment that can be offered to patients.

## Figures and Tables

**Table 1:** Medications and their doses from admission until the last follow-up visit. Gray cells indicate the titration of the beta-blocker therapy after the use of the Cardionica device.

		Medications					
Events	Days	ARB	ARNI	Beta-Blockers	Antialdosterones	Anticoagulants	Amiodarone
Admission and hospitalization (Cardiology)	1–15	Valsartan 80 mg/od	-	Bisoprololo 2.5 mg/od	Aldactone 50 mg/od	Apixaban 5 mg/bid	200 mg/od
Hospitalization (Rehabilitation)	15–30	-	Sacubitril/Valsartan 24/26 mg/bid	Bisoprololo 2.5 mg/od	Aldactone 50 mg/od	Apixaban 5 mg/bid	200 mg/od
Discharge	30	-	Sacubitril/Valsartan 24/26 mg/bid	Bisoprololo 2.5 mg/od	Aldactone 50 mg/od	Apixaban 5 mg/bid	200 mg/od
First follow-up visits	60		Sacubitril/Valsartan 24/26 mg/bid	Bisoprololo 2.5 mg/od	Aldactone 50 mg/od	Apixaban 5 mg/bid	200 mg/od
Additional visit—Cardionica prescription	120		Sacubitril/Valsartan 24/26 mg/bid	Bisoprololo 2.5 mg/od	Aldactone 50 mg/od	Apixaban 5 mg/bid	200 mg/od
Last follow-up visits	126		Sacubitril/Valsartan 24/26 mg/bid	Bisoprololo 5.0 mg/od	Aldactone 50 mg/od	Apixaban 5 mg/bid	200 mg/od

## Data Availability

The data presented in this study are available on request from the corresponding author. The data are not publicly available due to privacy restriction.
